# Floral micromorphology and transcriptome analyses of a fragrant Vandaceous Orchid, *Vanda* Mimi Palmer, for its fragrance production sites

**DOI:** 10.1186/s13104-017-2872-6

**Published:** 2017-11-02

**Authors:** Conie Toh, Ab. Rahim Mohd-Hairul, Nooraini Mohd. Ain, Parameswari Namasivayam, Rusea Go, Nur Ashikin Psyquay Abdullah, Meilina Ong Abdullah, Janna Ong Abdullah

**Affiliations:** 1 Department of Cell and Molecular Biology, Faculty of Biotechnology and Biomolecular Sciences, Universiti Putra Malaysia, 43400 UPM Serdang, Selangor Darul Ehsan Malaysia; 2 Faculty of Industrial Sciences and Technology, Universiti Malaysia Pahang, Gambang, 26300 Kuantan, Pahang Malaysia; 3 Institute of Bioscience, Universiti Putra Malaysia, 43400 UPM Serdang, Selangor Malaysia; 4 Department of Biology, Faculty of Science, Universiti Putra Malaysia, 43400 UPM Serdang, Selangor Malaysia; 5 Faculty of Agriculture and Food Sciences, Universiti Putra Malaysia Bintulu Sarawak Campus, 97008 Bintulu, Sarawak Malaysia; 6 Malaysian Palm Oil Board, 6 Persiaran Institusi, Bandar Baru Bangi, 43000 Kajang, Selangor Darul Ehsan Malaysia

**Keywords:** Fragrance, Monoterpene synthase, Morphology, Orchid, Stomata, Trichome

## Abstract

**Background:**

*Vanda* Mimi Palmer (VMP) is commercially valuable for its strong fragrance but little is known regarding the fragrance production and emission sites on the flowers.

**Results:**

Olfactory perception detected fragrance only from the petals and sepals. Light and Environmental Scanning Electron microscopy analyses on fresh tissues showed distributions of stomata and trichomes concentrated mostly around the edges. These results paralleled the rich starch deposits and intense neutral red stain, indicating strong fragrance and trichomes as potential main fragrance release sites. Next Generation Sequencing (NGS) transcriptomic data of adaxial and abaxial layers of the tissues showed monoterpene synthase transcripts specifically linalool and ocimene synthases distributed throughout the tissues. qPCR analyses taken at different time points revealed high levels of linalool and ocimene synthases transcripts in the early morning with maximal level at 4.00 am but remained low throughout daylight hours.

**Conclusions:**

Knowledge of the VMP floral anatomy and its fragrance production characteristics, which complemented our previous molecular and biochemical data on VMP, provided additional knowledge on how fragrance and flower morphology are closely intertwined. Further investigation on the mechanisms of fragrance biosynthesis and interaction of potential pollinators would elucidate the evolution of the flower morphology to maximize the reproduction success of this plant.

**Electronic supplementary material:**

The online version of this article (10.1186/s13104-017-2872-6) contains supplementary material, which is available to authorized users.

## Background

*Vanda* Mimi Palmer (VMP), a commercially viable orchid derived from the crossing of *Vanda* Tan Chay Yan and *Vanda tessellata* (Roxb.) Hk.*f*. ex G. Don, is endowed with the terrate-shaped flower of *Vanda* Tan Chay Yan as well as the tri-colour and strong floral scent of *Vanda tessellata*. Its sweet fragrance-producing ability earned it the Champion Award for Fragrant Orchid organized by the Royal Horticultural Society of Thailand in 1993 and the Best Orchid Fragrance in the 17th World Orchid Conference in 2002 [1]. VMP is popular in Malaysia, being mainly cultivated for potted-flower production.

Fragrance plays various functions in both floral and vegetative organs. Floral scent emission, in addition to colour, shape, surface structure and nectar guides, is a crucial strategy of plants to attract beneficial pollinating insects in ensuring reproduction [2–4]. Floral fragrances vary widely among species in terms of the number, identity, and relative amounts of constituent volatile compounds. Gas chromatography–mass spectrometry (GC–MS) analysis revealed that the VMP flower produced a fragrance composed of terpenoid, benzenoid and phenylpropanoid compounds [5].

Possible fragrance emission sites had been located at sites such as the flower epidermal cells for diffused emission [6], an enclosed area of the floral tissue specialized in scent emission termed as the osmophore by Vogel (as cited in [7]), glandular trichomes [6, 8–10] and floral stomata [11, 12]. Despite the commercial value of VMP due to its fragrance, little is known regarding both the fragrance production and the emission sites on the VMP flower. In our earlier work, we found fragrance transcripts and volatiles were actively produced in the flower [5, 13–17].

Hence, as a continuation of our earlier molecular work on fragrance, here we reported on the determination of the fragrance production and release sites by evaluating the histomorphological nature of the floral parts of VMP, and correlate them to the transcriptomic fluxes of the fragrance-related genes. To our knowledge, this is the first reported work on the floral anatomy and transcriptomes of this award winning fragrant orchid, VMP.

## Results and discussion

**Fig. 1:**
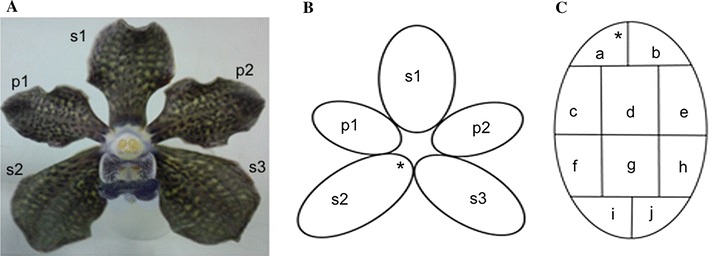
*Vanda* Mimi Palmer. **A** Flower. Scale bar = 6 mm; **B** Each floral part is designated as s1 = dorsal sepal, s2 = lateral sepal 1, s3 = lateral sepal 2, p1 = petal 1, p2 = petal 2. The labellum (L) is not included in the analysis; **C** For simplicity in presentation, each floral part is sectioned into 10 segments (a–j). Each segment was independently viewed under the Environmental Scanning Electron Microscope. Any trichome sitting on the border of the segments is not included in the count. The asterisk indicates the orientation of the segment (a) in the whole flower

**Fig. 2:**
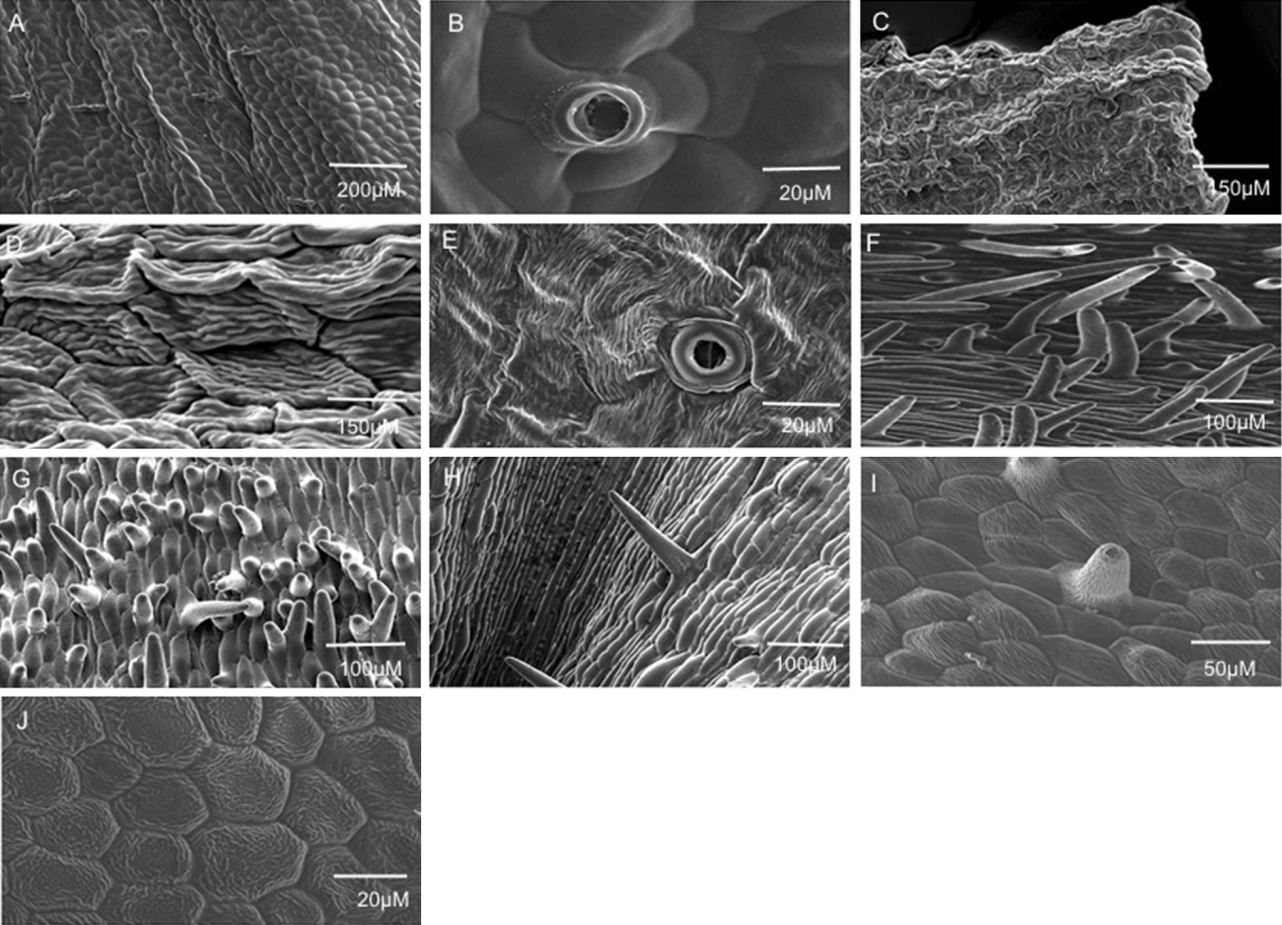
Surface morphology of *Vanda* Mimi Palmer floral parts. **A**, **B** stomata and trichomes on the sepal; **C**, **D** ridged adaxial epithelial cells on the labellum’s side-lobe; **E** stomata on the labellum; **F**, **G** dense protrusions/appendages that arise directly from the adaxial epidermal cells on the labellum’s mid-lobe; **H** long columnous adaxial epithelial cells on the labellum’s mid-lobe; **I** striated conical adaxial epithelial cells on the labellum’s mid-lobe; **J** polyhedral striated flattened adaxial epithelial cells

**Fig. 3:**
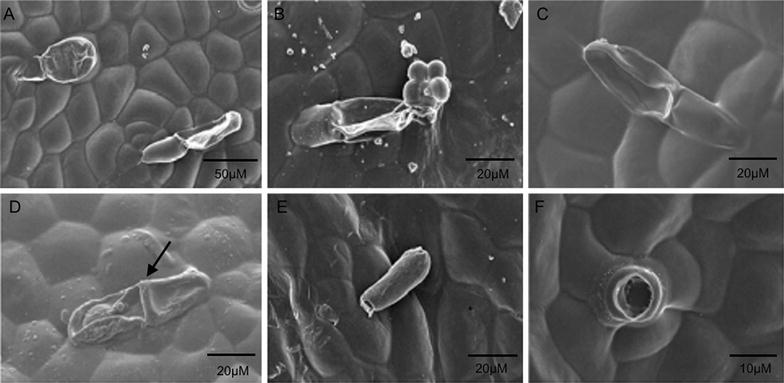
Features of glandular trichomes on petals and sepals of *Vanda* Mimi Palmer. **A**, **B** Trichomes easily collapsed. Note the basal cell numbers; **C** Note the club-shaped trichome; **D** Note the septum (arrow) separating the glandular cell and the stem cell; **E** Half-developed trichome found in the bud; **F** Gaping hole left behind after the trichome gets detached. **A**–**F** are developed trichomes found in partially-opened and completely-bloomed flower

**Fig. 4:**
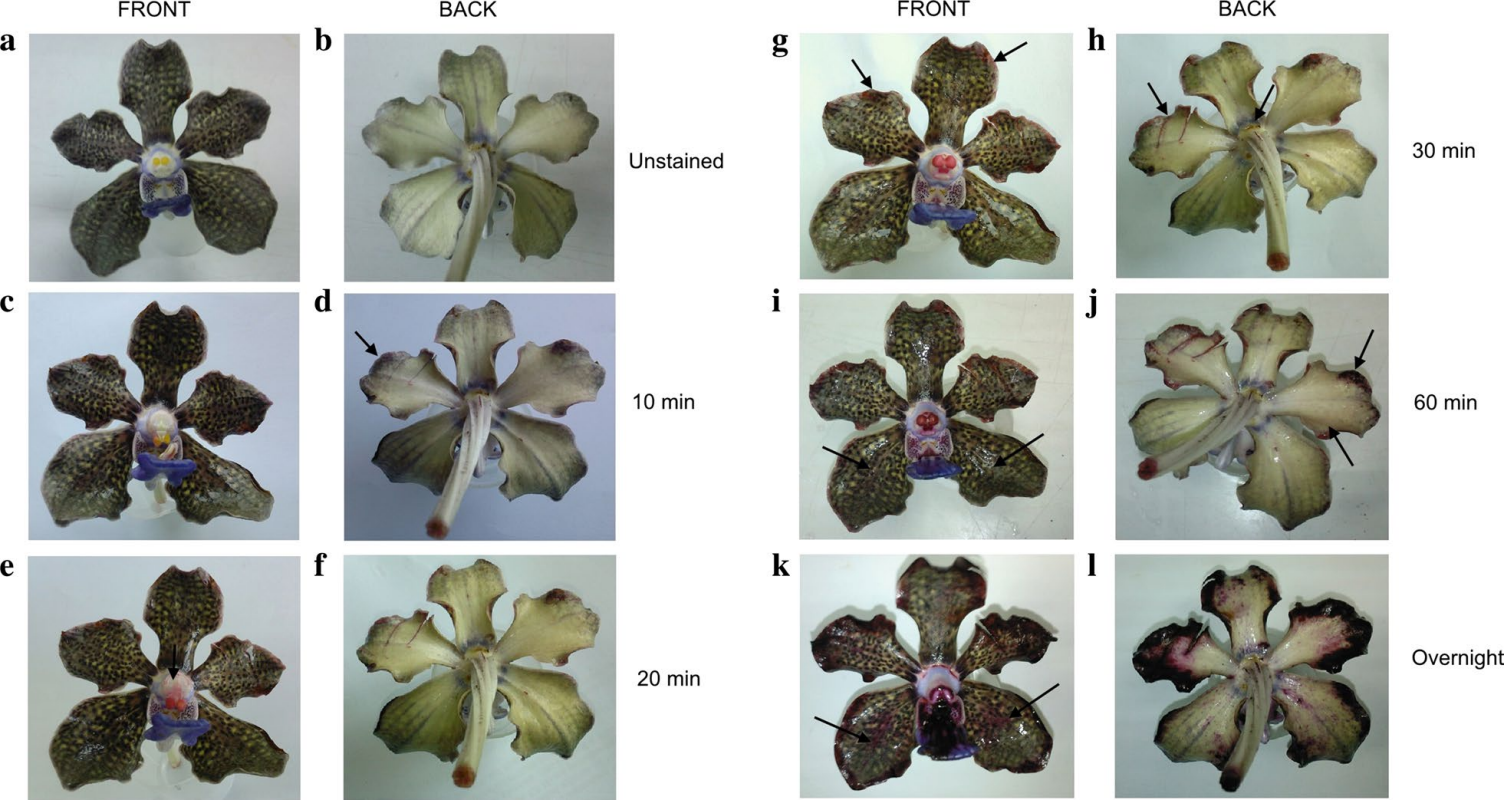
Neutral red staining of *Vanda* Mimi Palmer flowers. A whole flower was immersed in 0.5% (w/v) aqueous neutral red for 10 min to 24 h (overnight), and then rinsed in tap water. Arrows indicate the stained areas. FRONT indicates the frontal view of the flower, BACK indicates the dorsal view of the flower, **a** and **b** indicate unstained flower as control, **c** and **d** stained for 10 min, **e** and **f** stained for 20 min, **g** and **h** stained for 30 min, **i** and **j** stained for 60 min, and **k** and **l** stained overnight

Floral parts of VMP and their segmented areas are shown in Fig. 1 for floral surface analysis using SEM. It revealed that VMP epithelial cells of the petals and sepals were flat and interspersed with randomly distributed stomata and trichomes (Fig. 2A, B). The labellum’s side-lobe displayed ridged epithelial cells (Fig. 2C, D) and randomized stomata (Fig. 2E), and the mid-lobe contained dense protrusions/appendages (Fig. 2F, I) that arose directly from the epidermal cells, and a mixed morphology of epithelial cells such as long columnous cells (Fig. 2H), striated conical cells (Fig. 2I), and polyhedral striated flattened cells (Fig. 2J). Olfactory perception of the dissected floral parts (held one centimeter away) detected fragrance with equivalent intensities from the petals and sepals. However, no detectable fragrance was noted from the labellum. Trichomes present in VMP flower comprised of two unbranched types: glandular as found on the sepals and petals, and the non-glandular as found in the mid-lobe of the labellum. The non-glandular trichome retained its shape while the glandular trichome easily shrank when observed under the SEM (Fig. 2F, Fig. 3A, B). Generally, the labellum’s non-glandular trichome was longer than the glandular trichome (twice the length), was unbranched, single-celled, cylindrical, and arose directly from the epidermal floor (Fig. 2F–I). The glandular trichome was club-shaped (Fig. 3C) and comprised of two cells: a glandular cell supported by a stalk cell, which arose from varying numbers (4–6) of base cell. Both the glandular and stalk cells were almost equivalent in length (Fig. 3C). The glandular cell collapsed easily as compared to the stalk cell, which seemed hardier and succulent. Comparisons of the glandular trichomes from three flower developmental stages (bud, partially-opened flower, and completely-bloomed flower) revealed different features. Partially- and fully-opened flowers had well-developed, mature glandular trichomes. In the non-resupinated flower bud, some of the glandular trichomes observed on the sepals and petals were well-developed and some were under-developed. The partitioning of the two cells of the trichome was clearly seen on the well-developed trichomes (Fig. 3D) but not easily discernable on the under-developed ones (Fig. 3E). Both fully-opened flower and bud had well-developed glandular trichomes, respectively, but only fully-opened flower produced strong fragrance; hence, we postulated that the immaturity of the trichomes could be a factor in explaining why the bud was scentless. In our molecular findings, some of the fragrance-related transcripts were detected in the bud but preferentially at higher level in blooming flowers [13, 16]. There was no significant difference (*p* > 0.05) in terms of trichome density per 5 mm^2^ coverage area between petals and sepals (dorsal and lateral) from different developmental stages of the flower (Table 1). However, significant density differences (*p* < 0.05) were detected between the labellum and sepal/petal. Abundant cylindrical, single-celled trichomes were found on the labellum. Analyses of segmented petal and sepal revealed the edges of each segment (Fig. 1B, C) contained higher distribution of trichomes (data not shown). This finding paralleled the strong neutral red stain (Fig. 4) portrayed at the edges of each floral parts. Contrary to the distribution of trichomes, the stomata were randomly distributed over wider areas (Table 2) without any obvious concentration in any of the segments analysed. We postulated that the trichomes are more likely a site for fragrance release compared to the stomata.

**Table 1:** Comparison of the number of trichomes present at different floral developmental stages

Stage	Dorsal sepal	Lateral sepal	Petal	Labellum
Stage A	5 ± 2	6 ± 2	5 ± 1	20 ± 4^a^
Stage B	5 ± 2	6 ± 1	8 ± 3	16 ± 3^a^
Stage C	6 ± 1	6 ± 2	8 ± 3	18 ± 3^a^

*STAGE A* non-resupinated flower bud; *STAGE B* partially-opened resupinated flower; *STAGE C* completely-bloomed flower. Data are the means ± standard deviations of n = 24. Data was subjected to one-way analysis of variance (ANOVA). Multiple comparisons among means were performed using the Duncan’s multiple range test (DMRT) with significance level at p < 0.05

^a^Indicate means in the same row are significantly different

**Table 2:** The data was subjected to one-way analysis of variance (ANOVA), and the means compared using the Duncan’s multiple range test (DMRT) with significance level at p < 0.05

Floral parts	Mean stomata	Mean glandular trichomes
Dorsal sepal	121.00 ± 30.61^a^	60.68 ± 5.03
Lateral sepal 1	130.66 ± 12.66^a^	65.66 ± 2.87
Lateral sepal 2	122.66 ± 22.05^a^	73.67 ± 6.66
Petal 1	175.33 ± 6.11^a^	93.66 ± 1.53
Petal 2	163.66 ± 7.64^a^	77.99 ± 1.73

Data are the means ± standard deviations for each whorl

^a^Indicates means in the same column are significantly different

**Fig. 5:**
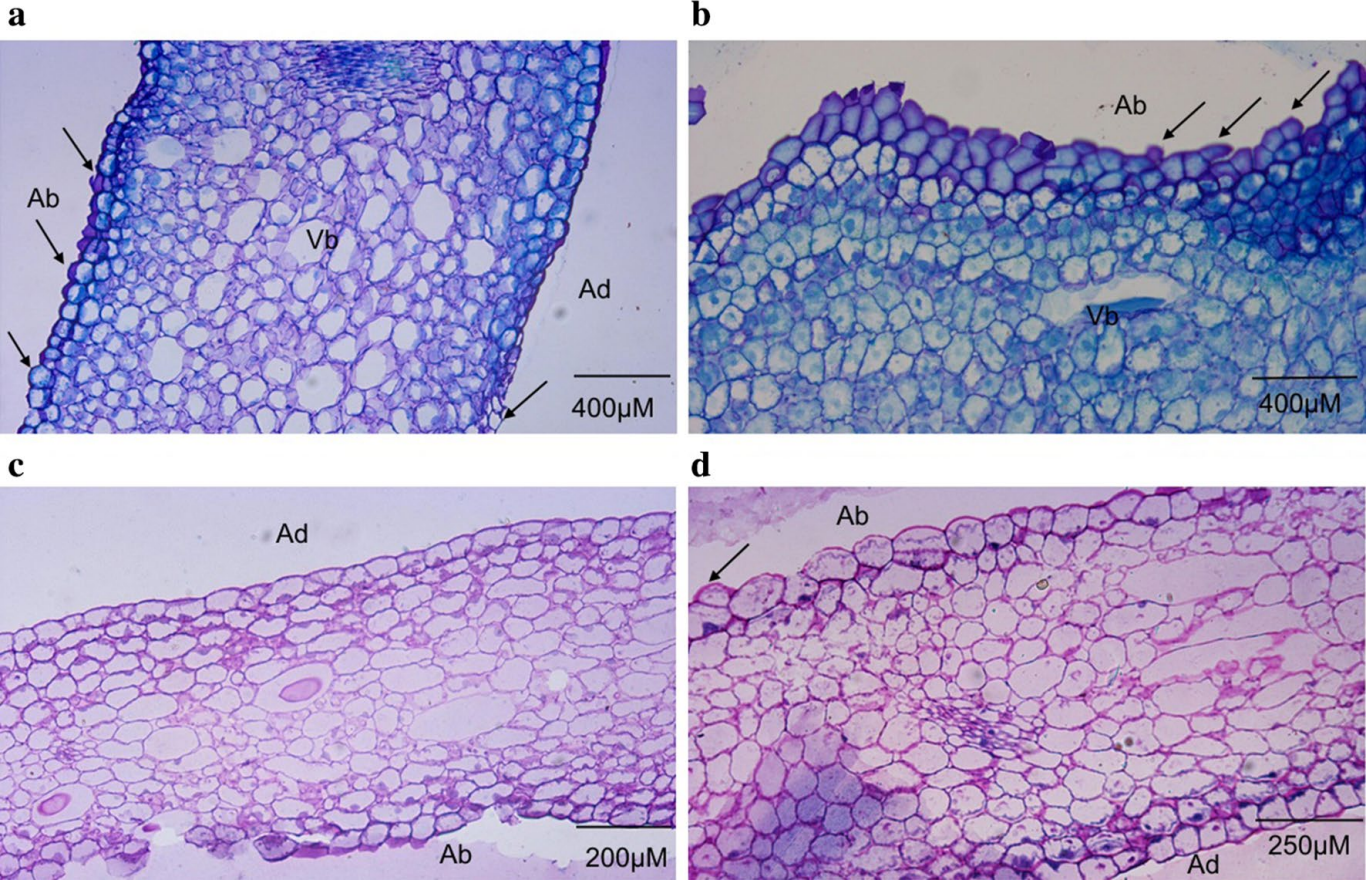
Photomicrographs of thin sections of *Vanda* Mimi Palmer petal and sepal. **a** and **b** are toluidine blue stained resin sections of a petal and sepal. **c** and **d** are resin sections stained with periodic acid-Schiff (PAS) and naphthol blue black. **a** Cross section of a sepal. The adaxial cells are frequently irregularly arranged compared to the regular arrangement of the abaxial cells. **b** Cross section of a petal. Like the sepal, the epidermal and sub-epidermal cells have big nuclei and dense cytoplasms (polysaccharides). Cross sections of petal (**c**) and sepal (**d**). The epidermal and sub-epidermal cells exhibit accumulation of polysaccharides and proteins. The parenchyma cells are mostly empty and loosely packed with large intercellular spaces. *Ab* Abaxial; *Ad* Adaxial; *Vb* Vascular bundles. Arrows indicate the trichomes (some intact, some broken)

Histochemical studies on VMP thin floral sections (5.0–7.0 µm) stained with toluidine blue (Fig. 5a, b) revealed accumulation of polysaccharides in the epidermal and sub-epidermal layers of both the sepal and petal tissues. Likewise, periodic acid-Schiff’s (PAS) reaction and naphthol blue black test revealed enormous deposits of polysaccharides and proteins present in the epidermal and sub-epidermal cell layers (Fig. 5c, d), suggesting both cell layers were active sites for metabolites production. The polysaccharides may be used as a source of energy or carbon for the biosynthesis of fragrant metabolites [15]. Quite notably is the fact that the adaxial epidermal cells are flat and regular, while the abaxial epidermal cells are flat but more irregular. Conical cells have been considered as a distinctive trait for petals that are found in majority of angiosperms and it is believed that the conical, papillate-shaped cells could enhance the diffusion of scent molecules or influence its directionality, besides enhancing light reflection, scent production and temperature [21–23], as well as reducing the flower wettability to increase the pollinator visitation rate [23]. The flatness observed was said to reflect less light, decrease the attractiveness of the flower and hence, attract less insects. It seems that the lack of physical attribute is compensated by the production of volatiles in this VMP to serve as attractant since VMP is often frequented by gnats.

Neutral red (NR) staining of detached fully-bloomed flowers was used for visualization of putative osmophores and as a first indication of the location of scent emission of VMP flower. Osmophores, floral tissues specialized for fragrance synthesis and secretion, were often found either at petal apices or on the adaxial surface of the dorsal sepal apex, or at both, depending on plant species [24]. We found that NR staining was positive in VMP, with more intense staining around the edges of the petals and sepals, compared to the rest of the floral tissues (Fig. 4). This also paralleled the density distribution of the trichomes on each floral parts examined (Table 2). The staining, which was faintly visible after 10 min in NR, intensified after 1 h and did not change significantly after 24 h. The results showed the permeability of the tissues to NR differed among different regions of the flower, and enhanced permeability could be caused by long-lasting storage ability of vacuoles that acted like a NR ion trap [6], or perhaps correlated with volatile emission. Thus, putative osmophoric flower parts or areas would take on the red stain. Hence, this implied that high accumulation of oil products might be present at the edges of the petals and sepals of VMP. This further suggested that both the epidermal layers of the petals and sepals were preferential sites of scent production and emission, as also reported in scented *Rosa* × *hybrida* petal [25] and supported by our transcriptomic data.

The sequencing data of the VMP petal-sepal transcriptome libraries of the adaxial and abaxial layers were submitted to the NCBI GenBank database (SRA Accession Numbers: SRR2064672, SRR2064673). The transcriptome analyses revealed the presence of 11,757 contigs in the pooled abaxial tissues and 17,555 contigs in the pooled adaxial tissues, covering a total of trimmed transcriptome data of 1.0 and 1.6 Gb, respectively. The gene ontology (GO) analysis on all the transcripts expressed in both the abaxial and adaxial tissues (Additional file 1) showed similar gene distribution patterns in both tissues for all three GO categories. Sequences of the targeted fragrance-related transcripts were submitted to the EMBL-EBI Database [PRJEB9553 (ERP010671)]. Their relative expressions profile (Additional file 2) showed the expressions of linalool synthase (contig 211), ocimene synthase (contig 1066), and sesquiterpene synthase (contigs 15, 58 and 81) transcripts were slightly lower in the adaxial layer compared to the abaxial layer. Phenylacetaldehyde synthase (contig 82, HO188241.1) that was previously reported to be up-regulated in VMP floral tissues and also developmentally regulated [14] was shown in this study to be up-regulated in the abaxial tissue and down-regulated in adaxial tissue with RPKM values of 4398 and 3888, respectively. Expressions of genes coding for the key regulatory enzymes of the fragrance biosynthetic pathways such as phenylalanine ammonia lyases (contigs 70 and 565) of the benzenoid and phenylpropanoid pathway, and HMG-CoA reductase (contig 144) that controls the mevalonate pathway were slightly up-regulated in the abaxial tissue.

**Fig. 6:**
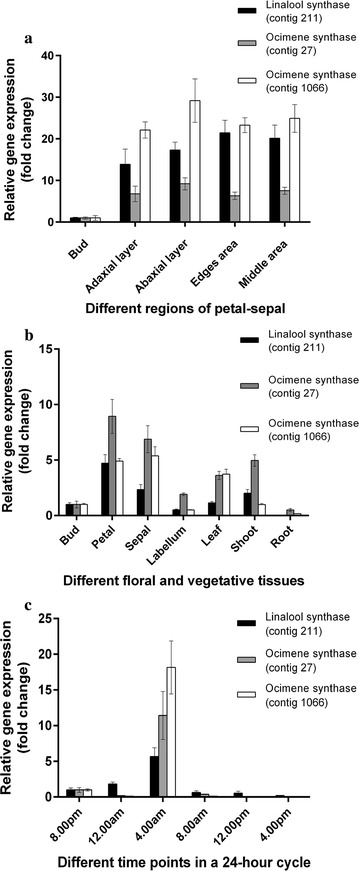
Expression analysis of monoterpene synthase transcripts in different tissues or parts of tissue, and at different time points in a 24-h cycle. **a** is comparative gene expression levels of linalool and ocimene synthases in different regions of petal-sepal, **b** is comparative gene expression levels of linalool and ocimene synthases in different floral and vegetative tissues, and **c** is comparative gene expression levels of linalool and ocimene synthases at different time points in a 24-h cycle

Figure 6a highlighted that three selected monoterpene synthase transcripts, linalool synthase (contig 211) and two ocimene synthases (contigs 27 and 1066), were expressed throughout the VMP petal and sepal in both the adaxial and abaxial layers. qPCR analysis on different floral tissues (bud, petal, sepal, and labellum) and three vegetative tissues (leaf, shoot and root) as summarized in Fig. 6b showed that linalool and ocimene synthases transcripts were up-regulated in the petal and sepal compared to the labellum and bud (calibrator tissue). The immature stage of VMP’s bud as previously reported [5] did not emit detectable fragrance compound until it achieved its fully-open flower stage. Such similar expression profiles were also obtained from our previous studies for phenylacetaldehyde synthase (VMPPAAS), sesquiterpene synthase (VMPSTS) and alcohol acyltransferase (VMPAAT) [5, 15, 16]. This observation also concurred with our findings of the high distribution of trichomes found at the edges of the petals and sepals, which might reflect their involvement in fragrance biosynthesis and emission. However, contrary to the floral tissues, the relative amount of the monoterpene synthase transcripts in the leaf and shoot were five-fold higher in comparison to the bud (calibrator tissue). In the current study, such contradictory levels of monoterpene synthase transcripts (Fig. 6b) present in the leaf and shoot, suggested that the vegetative tissues might also contribute to the biosynthesis of fragrance compounds. Unfortunately, the mode of transportation of the monoterpene compounds from the vegetative tissues via the phloem is still unclear in scented plants but it might be possible since methyl jasmonate, a volatile compound that is used for plant defense has been reported to be transported via vascular tissues [26]. In woody plants, monoterpene as well as sesquiterpene compounds in resin form have been reported to be synthesized in phloem-specialized tissues known as parenchyma cells as a response to attacks by fungi and bacteria [27]. This occurrence remains unknown for VMP.

Tissues sampling for fragrance-related studies in VMP had always been focused on specific time-points. Based on our previous studies on fragrance emission of VMP’s fully open flower [5], the emission of fragrance compounds including linalool and ocimene were detected as early as 6.00 a.m., reaching the highest level at 12.00–2.00 p.m., but reduced drastically after 4.00 p.m. We postulated that scent emission of floral tissues of VMP, especially in the petal and sepal, was closely associated with the effect of temperature whereby the highest level of sunlight coincided with the highest level of volatile emission while none of the fragrance compounds were detected at night probably due to the lower temperature as well as the inactivity of respective pollinators. Contrary to fragrance emission, the level of fragrance relative transcripts was much higher at night than during daytime (Fig. 6c) as observed also in our previous studies on VMP [14].

## Conclusions

The dense distribution of glandular trichomes on the adaxial part of the VMP petal and sepal, the active metabolic activities as well as transcriptomics profile in the epidermal cell layers of both the adaxial and abaxial parts of the petal and sepal highlight the significant contributions of those floral parts in fragrance compound biosynthesis, storage and emission. Overall, the morphohistological results suggest that trichomes are likely a key fragrance release site for VMP. However, the direct proof that the trichome is the vehicle for fragrance emission in VMP needs to be further confirmed with chemical analysis of extracts from the trichome itself. In addition, further investigations are needed to elucidate the mechanism and factors affecting fragrances biosynthesis besides their transportation mechanisms to the trichome as the fragrance release site.

## Methods

### Plant material

Mature *Vanda* Mimi Palmer (VMP) plants were bought and maintained at the United Malaysian Orchids Nursery in Rawang, Selangor, Malaysia. The orchids were grown in pots filled with charcoal, and the pots were placed in an open environment under shade of approximately 70–80% sunlight exposure and temperature of between 25 and 30 °C. Unless otherwise stated, freshly opened flowers of VMP were used for the histochemical staining and assays, and SEM analyses. Only freshly opened flowers produced strong fragrance while the buds were scentless [13, 15, 18]. The flower (Fig. 1A) was divided into three main sections, namely labellum (modified petal), sepal (dorsal and lateral), and petal. All the floral parts were cut into small pieces measuring 5 mm^2^, and fixed for subsequent analyses.

### Floral surface analysis

Surface morphology analysis was carried out using a DSM960A Scanning Electron Microscope (Carl Zeiss, Germany). Samples for surface analysis were fixed using FAA solution (10% formalin solution, 20% acetone and 70% absolute alcohol, with an additional one drop of glycerol) and Copenhagen solution (1% glycerol, 29% of distilled water and 70% of methyl-spirit alcohol) to preserve the specimens. Dehydration was done in an alcohol dilution series (30, 50, 70, 90, 95%, followed by twice in 100%). Samples were critical point dried and sputter-coated with gold–palladium using conventional protocols, and then viewed using the electron microscope. Quantitative density estimations of the trichome distributions over a 5 mm^2^ area were obtained by viewing the adaxial floral parts (petals, sepals and labellum) of three different developmental stages of the VMP flowers (*STAGE A*: non-resupinated flower bud, *STAGE B*: partially-opened resupinated flower, and *STAGE C*: completely-bloomed flower) under the same magnification. The trichome counts were taken from a total of 24 biological replicates for each floral part. Quantitative estimations of trichome and stomata distributions on each adaxial perianth (sepal *versus* petal) were determined (see Fig. 1A–C for the schematic partitioning of the areas analyzed). Only trichomes falling within each selected segment were taken into consideration for enumeration. Three biological samples which consisted of three different sepals and three different petals from completely-bloomed flowers formed on the same inflorescence were analyzed. All quantification experiments were performed in a completely randomized design (CRD). The data were subjected to one-way analysis of variance (ANOVA) using the SPSS version 16 software (SPSS, Chicago, IL). Multiple comparisons among means were performed using the Duncan’s multiple range test (DMRT) with the level of significance at *p* < 0.05.

### Histological analysis

The histological procedures were done according to the protocol adapted from the ORSTOM-CIRAD Team of France. The samples were fixed with 25% glutaraldehyde-paraformaldehyde-caffeine fixing solution for 24–48 h at room temperature (26 ± 1 °C) followed by dehydration in a series of ethanol: 30% for 30 min, 50% for 45 min, 70% for 45 min, 80% for 60 min, 90% for 60 min, 95% for 60 min, and twice in absolute ethanol for 60 min each. The samples were further bathed in butanol three times for a minimum of 24 h per bath, and then in impregnation-butanol solution (1:1, v:v) for a minimum of 48 h. The samples were then infiltrated in resins according to the manufacturer’s instructions (Technovit Histo-Technique 7100, Heraeus Holding GmbH, Hanau, Germany) for 24 h at 4 °C followed by the embedding step in impregnation-hardener II solution (15:1, v:v), where the samples were left to dry in the mold overnight. Sections of 5.0–7.0 µm were cut using a Leica RM 2165 microtome (Leica Microsystems, Nussloch, Germany) and mounted on glass slides. The specimens were then gently heated at 60 °C on a slide drying bench (Electrothermal—Thermo Scientific MH6616, UK). Periodic acid-Schiff (PAS) reaction and naphthol blue black test were carried out to check for the presence of polysaccharides and proteins, respectively. The specimen slides were soaked in 1% (v/v) periodic acid for 5 min, and then rinsed four times in distilled water at pH 4.5. This was followed by soaking in Schiff reaction solution for 20 min in the dark and rinsing four times in distilled water at pH 4.5. Next, the slides were soaked in naphthol blue black solution at 60 °C for 5 min and thoroughly rinsed three times in distilled water. Finally, the slides were mounted with Surgipath mounting medium inside a fume hood and dried for 1 day prior to examination. Oil gland structures were investigated by staining with toluidine blue O at pH 5.6, a general stain for tissue structure [9, 19]. The specimen slides were soaked in 0.1% (v/v) toluidine blue O solution for 1 min and rinsed in distilled water for 2–3 min. The slides were then mounted with Surgipath mounting medium inside a fume hood and dried for 1 day prior to examination. Neutral red staining [6] was carried out to detect the presence of osmophores in the floral organs. A whole flower was immersed in 0.5% (w/v) aqueous neutral red for 30 min to 24 h. After staining, the flower was rinsed in tap water and photographed. All histological samples were examined, viewed and photographed with a camera attached to the LEICA DM6000 B light microscope (Leica, Germany) and processed using the Progress Research Pro software (Leica, Germany).

### Construction and analysis of petal-sepal transcriptome libraries

The petals and sepals were collected from full bloom flowers of VMP and pooled as one tissue source (designated as petal-sepal). The adaxial and abaxial layers of both tissues were separated equally using a scalpel blade, and immediately placed on ice to prevent any RNA degradation. Two grams of each pooled adaxial and abaxial layers were subjected to total RNA extraction as described by [13]. PolyA + mRNAs were isolated from total RNA of each adaxial and abaxial layer, separately using the PolyATract^®^ mRNA Isolation System III (Promega, USA) following the manufacturer’s instructions. A total amount of 50 ng of polyA + mRNA of each pooled adaxial and abaxial layers was subjected to transcriptome library construction using the ScriptSeq™ v2 RNA-Seq kit (Epicentre, a Illumina company, USA). The transcriptome libraries were sent to Science Vision Sdn. Bhd. (Selangor, Malaysia) for sequencing using the MiSeq Desktop Sequencer (Illumina, USA).

### Analysis of the petal-sepal transcriptome libraries

Forward and reverse sequences from each petal-sepal adaxial and abaxial transcriptome libraries were paired together with acceptable paired reads of between 100 and 250 bp, and trimmed to remove adaptors and low quality sequences using the CLC Genomic Workbench software (CLC Bio, a Qiagen company, Denmark). All the trimmed sequences of each library were assembled using the de novo assembly tool in the CLC Genomic Workbench software to create simple contig sequences. The contig sequences were then subjected to BLASTX analysis using the Blast2go Pro software (BioBam Bioinformatics, Spain) run on JAVA platform on Windows 7 operating system, and functionally characterized by mapping with the GO (Gene Ontology) databases. Mapped sequences were annotated and visualized for gene distribution based on their biological processes, cellular components and molecular functions. Expression analyses were carried out by comparing the expression levels of selected fragrance-related transcripts in both transcriptome libraries using the CLC Genomic Workbench Software (CLC Bio, a Qiagen company, Denmark). Raw data of both transcriptome libraries were trimmed for adaptor as well as low quality sequences before being subjected to de novo assembly, separately, using the CLC Genomic Workbench software. All contig sequences from both adaxial and abaxial transcriptomes were then further re-assembled. Contig sequences retrieved from the second de novo assembly of the pooled two transcriptomes were used as a ‘reference genome’ for expression analysis due to unavailability of a full genome sequence of VMP. An experiment was setup in the CLC Bio software using a ‘two-group’ comparison approach to compare the expressions of selected fragrance-related transcripts in the adaxial and abaxial layers. In the experiment, trimmed petal-sepal adaxial and abaxial transcriptome raw data were mapped with the ‘reference genome’. The original expression value of each selected fragrance-related transcript was normalized using a scaling approach available in the CLC Genomics workbench software whereby each set of sample’s expression values was multiplied by a constant to normalize the sample’s value against the same ‘target’ value. The normalized expression value of each gene was subjected to statistical analysis, based on proportion, using Kal’s test with Befferoni corrected *p* values.

### Expression analyses of monoterpene synthase transcripts using real-time RT-PCR

Expression analyses of a linalool synthase (contig 211) and two ocimene synthase (contigs 27 and 1066) transcripts were carried out using real-time RT-PCR for different tissues or parts of a tissue as well as at different time points in a 24-h cycle. Samples from different selected areas of the petal and sepal including adaxial layer, abaxial layer, middle area and edges as well as specific floral and vegetative tissues including bud, petal, sepal, labellum, leaf, shoot and root were collected at 4.00 am. Meanwhile, for expression studies at different time points in a 24-h cycle, petals and sepals were collected at 4-h intervals (8.00 p.m., 12.00 a.m., 4.00 a.m., 8.00 a.m., 12.00 p.m. and 4.00 p.m.). In this study, total RNA was extracted from all the collected samples according to the method described by [13]. The extracted total RNA for each sample was used for reverse transcription into first-strand cDNA using the QuantiTect^®^ Reverse Transcription Kit (Qiagen, USA) according to the manufacturer’s instructions. The synthesized first-strand cDNA of each sample was used as a template for the expression analysis of the monoterpene synthases transcripts using the Mastercycler^®^ ep Realplex Real-time PCR (Eppendorf, Germany), together with the housekeeping genes as endogenous controls (actin, AF246716.1; alpha tubulin, GW687608.1; cyclophilin, GU942927.1). The real-time RT-PCR reactions were carried out in three replicates for each sample in a total volume of 20 µL containing 1X SYBR^®^ Green I Hot-start Real-time PCR mix (GeneCraft, Germany), 1 µL of fivefold diluted first-strand cDNA template and 150 nM of gene-specific primers using the following program: 95 °C for 2 min; 40 cycles of 95 °C for 30 s, annealing at 56 °C for 30 s and extension at 72 °C for 30 s; 81 cycles for melting curve analysis; and 10 s for each 0.5 °C (55–95 °C). The expression profiles of the monoterpene transcripts in different tissues or parts of a tissue, and at different time points in a 24-h cycle were constructed by using the ΔΔC_T_ comparative method [20] and normalized with the expression of all the endogenous controls.

## Additional files

**Additional file 1.** Gene ontology (GO) comparison between (A) adaxial layer and (B) abaxial layer of *Vanda* Mimi Palmer’s petal-sepal.

**Additional file 2.** Differential Expression analyses of fragrance-related genes between adaxial and abaxial layers of *Vanda* Mimi Palmer’s petal-sepal based on transcriptomic sequencing data.

## Abbreviations

VMP: *Vanda* Mimi Palmer
SEM: Scanning Electron Microscope
PAS: periodic acid-Schiff’s
RPKM: Reads Per Kilobase per Million mapped reads
HMG-CoA: 3-hydroxy-3-methyl-glutaryl-coenzyme A
RNA: ribonucleic acid
