# Supplementary figures and images for: Floral micromorphology and transcriptome analyses of a fragrant Vandaceous Orchid, Vanda Mimi Palmer, for its fragrance production sites
Source: BMC Res Notes. 2017 Nov 2;10:554. doi: 10.1186/s13104-017-2872-6 (PMC5669028; doi:10.1186/s13104-017-2872-6)

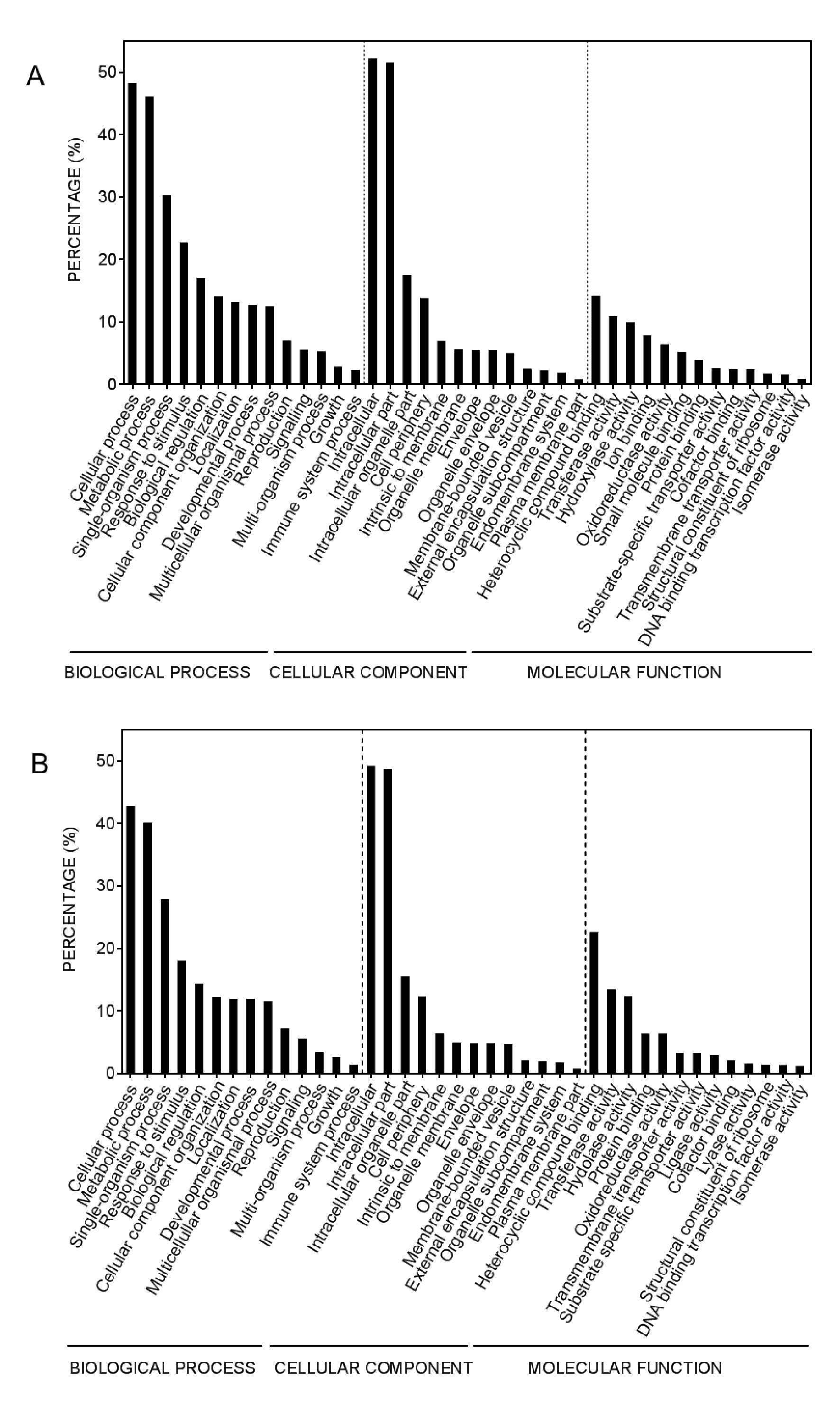

Supplement: Supplementary file 1 — Additional file 1. Gene ontology (GO) comparison between (A) adaxial layer and (B) abaxial layer of Vanda Mimi Palmer’s petal-sepal. [file 13104_2017_2872_MOESM1_ESM.jpg]
