# Supplementary material for: Floral micromorphology and transcriptome analyses of a fragrant Vandaceous Orchid, Vanda Mimi Palmer, for its fragrance production sites
Source: BMC Res Notes. 2017 Nov 2;10:554. doi: 10.1186/s13104-017-2872-6 (PMC5669028; doi:10.1186/s13104-017-2872-6)
Supplement: Supplementary file 2 — Additional file 2. Differential Expression analyses of fragrance-related genes between adaxial and abaxial layers of Vanda Mimi Palmer’s petal-sepal based on transcriptomic sequencing data. [file 13104_2017_2872_MOESM2_ESM.docx]

| Transcript/gene name | Gene Length  (bp) | Adaxial layer | | Abaxial layer | | Kal's test (Z-test) | |
| --- | --- | --- | --- | --- | --- | --- | --- |
|  |  | Total  Reads | RPKM | Total  Reads | RPKM | Fold change | P-value |
| Linalool synthase (contig 211) | 2416 | 177 | 337.00 | 345 | 462.23 | 1.372 | 0.00042 |
| Nerolidol synthase (contig 397) | 752 | 24 | 146.81 | 30 | 129.13 | -1.136 | 0.639159 |
| Nerolidol synthase (contig 398) | 773 | 83 | 493.91 | 101 | 422.94 | -1.168 | 0.29052 |
| Nerolidol synthase (contig 760) | 887 | 6 | 31.12 | 8 | 29.19 | -1.065 | 0.905625 |
| Ocimene synthase (contig 27) | 2006 | 26 | 59.62 | 81 | 130.70 | 2.192 | 0.000189 |
| Ocimene synthase (contig 1066) | 2435 | 54 | 102.01 | 92 | 122.30 | 1.198 | 0.280807 |
| Sesquiterpene synthase (contig 15) | 931 | 3595 | 17762.40 | 5395 | 18757.55 | 1.056 | 0.009334 |
| Sesquiterpene synthase (contig 81) | 437 | 642 | 6757.81 | 1173 | 8688.62 | 1.286 | 1.36E-07 |
| Phenylacetaldehyde synthase (contig 82) | 2132 | 1802 | 3887.94 | 2897 | 4398.41 | 1.131 | 2.48E-05 |
| Pheylalanine ammonia lyase (contig 70) | 2275 | 627 | 1267.76 | 549 | 1545.19 | 1.219 | 5.12E-05 |
| Pheylalanine ammonia lyase (contig 435) | 2590 | 10 | 17.76 | 7 | 8.75 | -2.042 | 0.144842 |
| Pheylalanine ammonia lyase (contig 565) | 2604 | 635 | 1121.72 | 980 | 1218.20 | 1.086 | 0.098503 |
| HMGR (contig 144) | 2089 | 469 | 1032.73 | 881 | 1365.12 | 1.322 | 4.85E-07 |
